# Multi-Omics Revealed Molecular Mechanisms Underlying Guard Cell Systemic Acquired Resistance

**DOI:** 10.3390/ijms22010191

**Published:** 2020-12-27

**Authors:** Lisa David, Jianing Kang, Daniel Dufresne, Dan Zhu, Sixue Chen

**Affiliations:** 1 Department of Biology, University of Florida, Gainesville, FL 32611, USA; lisaidavid@ufl.edu (L.D.); jianing.kang@ufl.edu (J.K.); zhudan2014dora@163.com (D.Z.); 2 Genetics Institute (UFGI), University of Florida, Gainesville, FL 32610, USA; 3 College of Life Science, Northeast Agricultural University, Harbin 150030, China; 4 Department of Chemistry, Florida Atlantic University, Boca Raton, FL 33431, USA; dufresne71@yahoo.com; 5 Key Lab of Plant Biotechnology in Universities of Shandong Province, College of Life Science, Qingdao Agricultural University, Qingdao 266109, China; 6 Plant Molecular and Cellular Biology Program, University of Florida, Gainesville, FL 32610, USA; 7 Proteomics and Mass Spectrometry, Interdisciplinary Center for Biotechnology Research (ICBR), University of Florida, Gainesville, FL 32610, USA

**Keywords:** systemic acquired resistance, guard cell, priming, multi-omics, stomatal immunity

## Abstract

Systemic Acquired Resistance (SAR) improves immunity of plant systemic tissue after local exposure to a pathogen. Guard cells that form stomatal pores on leaf surfaces recognize bacterial pathogens via pattern recognition receptors, such as Flagellin Sensitive 2 (FLS2). However, how SAR affects stomatal immunity is not known. In this study, we aim to reveal molecular mechanisms underlying the guard cell response to SAR using multi-omics of proteins, metabolites and lipids. *Arabidopsis* plants previously exposed to pathogenic bacteria *Pseudomonas syringae pv*. tomato DC3000 (*Pst*) exhibit an altered stomatal response compared to control plants when they are later exposed to the bacteria. Reduced stomatal apertures of SAR primed plants lead to decreased number of bacteria in leaves. Multi-omics has revealed molecular components of SAR response specific to guard cells functions, including potential roles of reactive oxygen species (ROS) and fatty acid signaling. Our results show an increase in palmitic acid and its derivative in the primed guard cells. Palmitic acid may play a role as an activator of FLS2, which initiates stomatal immune response. Improved understanding of how SAR signals affect stomatal immunity can aid biotechnology and marker-based breeding of crops for enhanced disease resistance.

## 1. Introduction

Systemic acquired resistance (SAR) is an inducible defense mechanism that is activated throughout the plant after a localized pathogen infection. It confers resistance to a broad range of pathogens in systemic tissues. SAR occurs when the plant delivers mobile signals from the primary site of infection to the remote, uninfected tissues [1,2,3,4,5]. Upon receiving the mobile immune signals, the remote tissue mounts a defense against the pathogens in a robust manner. This mechanism is called “priming” and those uninfected remote leaves are said to be “primed” for pathogen response. SAR priming endows the plant with a broad-spectrum resistance, including to pathogens that cause cell death and tissue necrosis [6,7,8]. The SAR molecular mechanisms may be evolutionarily conserved and are found in a wide range of dicotyledon and monocotyledon plant species [9]. Upon pathogen attack, plant pattern recognition receptors (PRRs), first identified in rice and *Arabidopsis* (XA21 and FLS2, respectively), recognize conserved pathogen-associated molecular patterns (PAMPs) (e.g., flagellin peptide), and activate PAMP-triggered immunity (PTI) [10,11,12]. Plants also contain immune receptors that recognize a great variety of pathogen effectors via nucleotide-binding site and leucine-rich repeats (NBS-LRR) proteins to induce effector triggered immunity (ETI) [10]. Activation of SAR in systemic tissue is associated with increased transcript abundance of pathogenesis-related (PR) genes and accumulation of a defense hormone salicylic acid (SA) [13]. In *Arabidopsis thaliana*, the receptor for SA during SAR has been identified as the protein non-expressor of pathogenesis related-1 (NPR1), a master regulator in SAR [6,14,15,16,17].

SAR response is typically studied on a whole-plant or whole-leaf level, masking specific responses of different cell types. Guard cells form stomata on leaf surfaces, and their primary function is to open and close the stomata for gas exchange. Guard cells are highly responsive to a number of environmental cues, such as atmospheric CO_2_ concentration, blue and red light, and pathogens [18,19,20,21]. Initial work from Melotto et al. [20] showed that guard cells close stomata upon perception of bacteria, and that a pathogenic bacterium *Pseudomonas syringae pv* tomato (*Pst*) DC 3000 was able to re-open the closed stomata. *Pst* secretes a toxin called coronatine (COR), which is a chemical mimic of the active form of the plant hormone jasmonate-isoleucine (JA-Ile). COR binds to the JA-Ile receptor coronatine-insensitive 1 (COI1) and initiates a signal cascade triggering guard cells to open stomata [22,23,24].

Plant reactive oxygen species (ROS) signaling and redox-regulated proteins in SAR have been reported in a number of publications, e.g., [25,26,27,28,29,30,31]. The roles of ROS in plant immune responses have been summarized in recent reviews [32,33]. ROS can serve as signaling molecules that are needed for activation of defense response. Toum et al. [34] found that COR inhibits nicotinamide adenine dinucleotide phosphate (NADPH) oxidase-dependent ROS production and stomatal closure induced by abscisic acid (ABA), the flagellin peptide flg22, and darkness. However, COR did not prevent SA-induced ROS production through peroxidases [34]. As SAR increases SA concentrations in cells of primed leaves, it would be reasonable to predict that the increased SA in guard cells could induce ROS production, leading to an altered stomatal immune response to COR. Different classes of lipids are involved in plant defense and ROS signaling. For example, galactolipids monogalactosyldiacylglycerol (MGDG) and digalactosyldiacylglycerol (DGDG) play a role in regulating cellular ROS levels [35]. During SAR, DGDGs contribute to the nitric oxide (NO) cellular pool and induce SA biosynthesis. In addition, MGDGs regulate the biosynthesis of the SAR signals azelaic acid (AzA) and glycerol-3-phosphate (G3P) downstream of NO [35]. AzA is derived from the oxidation of C18 unsaturated fatty acids (FAs), a process activated by ROS. The NO-ROS-AzA-G3P signaling operates in parallel with SA to activate SAR [35].

Here we aim to identify specific SAR responses in guard cells and putative molecular mechanisms underlying the guard cell SAR response. We report stomatal movement and guard cell molecular changes underlying stomatal immune responses to SAR. Through multi-omics of proteins, metabolites, and lipids from guard cells of uninoculated leaves of primed *Arabidopsis* plants, we found that ROS and FA signals are activated in the primed guard cells, and we also identified proteins, lipids, and metabolites with previously unknown functions, which may be involved in the systemic stomatal immune response.

## 2. Results

### 2.1. Stomatal Movement in Response to Priming Correlates with Reduced Bacterial Colonization

**Figure 1:**
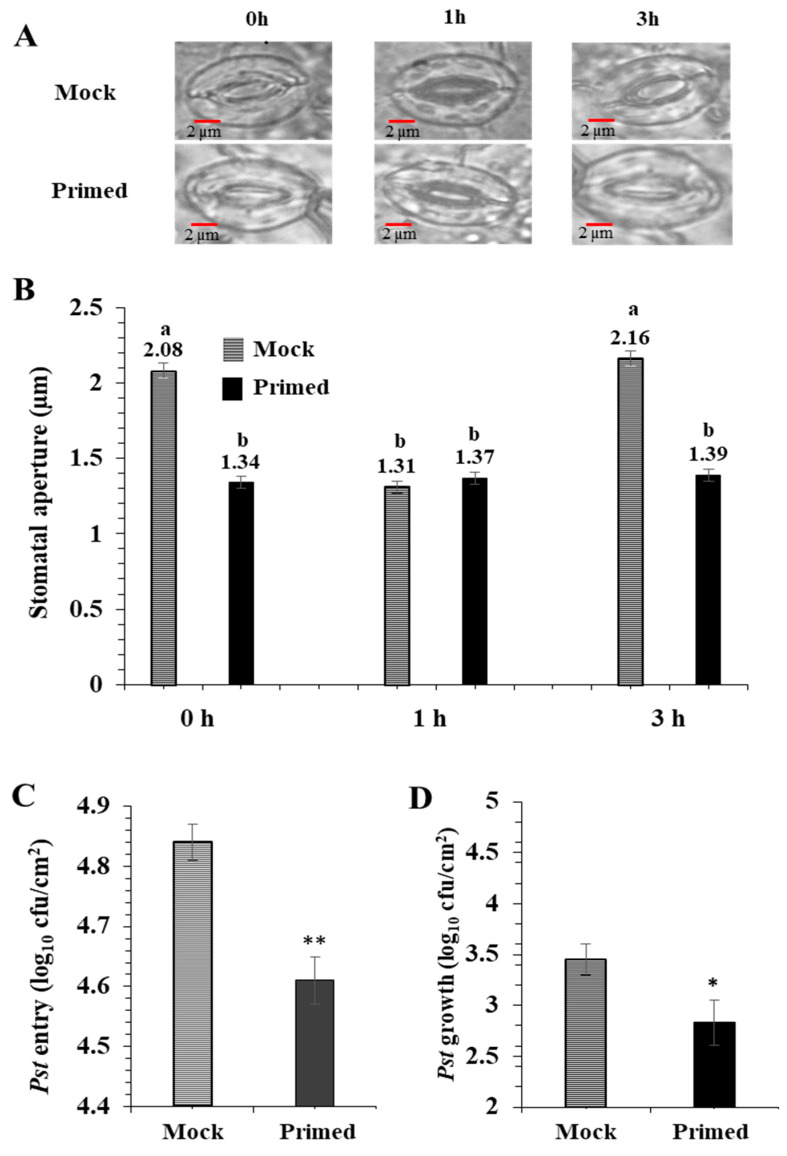
Pathogen entry and growth assays revealed enhanced stomatal immunity in primed *Arabidopsis* leaves. (**A**) Images showing representative stomatal apertures in mock and primed *Arabidopsis* leaves after 0, 1, and 3 h after secondary exposure to *Pst* DC3000. (**B**) Quantitative measurements of 150 stomata from three replicate experiments. Statistically significant differences were marked by a and b. (**C**) *Pst* DC 3000 entry results obtained from nine biological replicates of primed and mock plants. The data are presented as average ± standard error. (**D**) *Pst* DC 3000 growth results obtained from nine biological replicates of primed and mock plants. The data are presented as average ± standard error. ** *p* < 0.01, * *p* < 0.05.

For the priming experiments, *Pst* (0.02 OD600 in 10 mM MgCl_2_) or 10 mM MgCl_2_ (mock) was infiltrated into one mature rosette leaf. After 72 h, the opposite rosette leaf was exposed to the *Pst* (0.2 OD600). As previously reported by Melotto et al. [20], the basal immune response of the mock-treated stomata closed after 1 h exposure to *Pst,* and then re-opened after 3 h. Surprisingly, the primed leaves did not exhibit such stomatal immune responses (Figure 1A). They maintained a small stomatal aperture during the entire period of *Pst* exposure. There was no significant difference in the stomatal aperture from the primed leaves taken at 0, 1, and 3 h after *Pst* exposure (Figure 1B). It can be noted that due to the perception of PAMPs, the 1 h mock and primed apertures are similar. To examine if the stomatal apertures affected bacterial entry into the leaf apoplastic space, we conducted bacterial entry assays. As expected, after 3 h exposure to *Pst*, significantly more bacteria entered the mock leaves compared to the primed leaves (Figure 1C). To examine overall susceptibility to *Pst*, we measured bacterial growth in the leaves. Significantly more bacteria colonized the mock leaves than the primed leaves. (Figure 1D).

### 2.2. Differentially Abundant Proteins in the Primed and Mock Guard Cells

**Figure 2:**
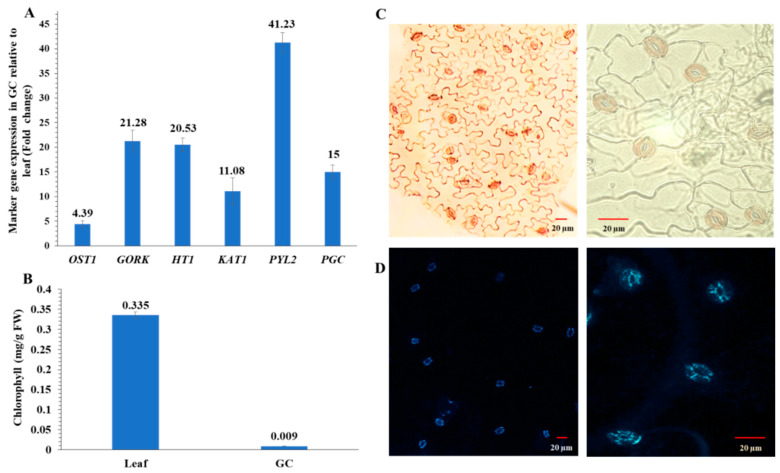
Enriched guard cell (GC) purity and viability assays. (**A**) Transcript abundances of six genes based on their fold differences in guard cell samples compared to leaf samples. Standard error of the mean was calculated from three biological replicates and three technical replicates. OST1, open stomata 1; GORK, guard cell outward-rectifying potassium channel; HT1, high temperature 1; KAT1, potassium channel; PYL2, pyrabactin resistance-like 2; and PGC, promoter guard cell 1 (At1g22690). (**B**) Chlorophyll contents in four replicates of leaves or enriched guard cells. The data are presented as average ± standard error. (**C**) Neutral red staining of the enriched guard cells. (**D**) Fluorescein diacetate (FDA) staining of the enriched guard cells isolated with a blender.

To ensure that guard cell samples used for multi-omic experiments were highly enriched, we conducted a purity assay of the guard cell samples using RT-qPCR with primers for guard cell-specific transcripts. Relative expression levels of these genes were very high (Figure 2A). Purity was further confirmed by low chlorophyll levels in the guard cell samples compared to the leaves (Figure 2B). In addition, guard cells appeared viable based on neutral red (Figure 2C) and fluorescein diacetate (FDA) staining (Figure 2D).

**Figure 3:**
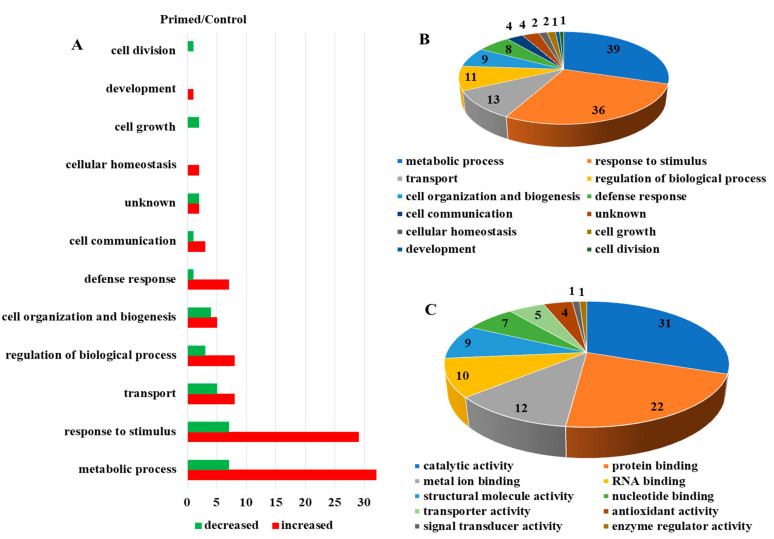
Dynamic protein changes in primed guard cells and functional classification. (**A**) Numbers of proteins with altered abundances in primed *Arabidopsis* guard cells categorized by biological function. (**B**) Biological processes of the identified guard cell proteins. (**C**) Molecular functions of identified guard cell proteins.

Proteomic analysis of mock-treated versus primed guard cell samples taken from uninoculated leaves after *Pst* treatment identified 587 proteins, each with more than one unique peptide (1% FDR) (Table S1). Of the identified proteins, 55 showed differential abundances in the primed guard cells compared to the mock guard cells (Figure 3A). Two large groups of the differential proteins (mostly increased after priming) were related to metabolic processes (39 proteins) and responses to stimuli (36). Transport proteins (13), regulation of biological processes (11), and cell organization and biogenesis (9) were also increased overall in the primed guard cells compared to the mock samples. In addition, defense response (8 proteins), and cell communication (4) also mostly increased in the primed guard cells (Figure 3B). The molecular functions of these proteins were categorized and the most abundant groups were catalytic activity (31), protein binding functions (22), metal binding functions (12), and RNA binding proteins (10). Other protein functions included proteins with structural molecule activity (9) and transporter activity (5) among the differential proteins related to priming (Figure 3C). Of these differential proteins, 27 were mapped to Kyoto Encyclopedia of Genes and Genome (KEGG) pathway. Interestingly, four glutathione-S-transferases (GSTU19, GSTF9, GSTF7, and GSTL3) were all increased in the primed guard cells compared to the mock. Also of interest was the identification of a SA-related legume family protein that was increased about two-fold in the primed guard cells. In addition, the following three proteins showed largest increases in the primed guard cells compared to the mock (Table 1): an endoribonuclease family protein involved in cellular response to stimulus, a rotamase cytochrome P(CYP) 3 (ROC3), and a GST F7 in glutathione metabolism related to defense responses to bacteria and fungi (Table 1). Furthermore, three proteins were only identified in the primed guard cells: a glycosyl transferase family 35 protein (PHS1) and a calcium-dependent lipid-binding (CaLB domain) protein involved in response to stimulus (such as hypoxia stress), and a stress-responsive Tudor-SN protein 2 (Tudor2) with RNA binding activity (Table S1).

### 2.3. Differential Metabolites in the Primed and Mock-treated Guard Cells

**Figure 4:**
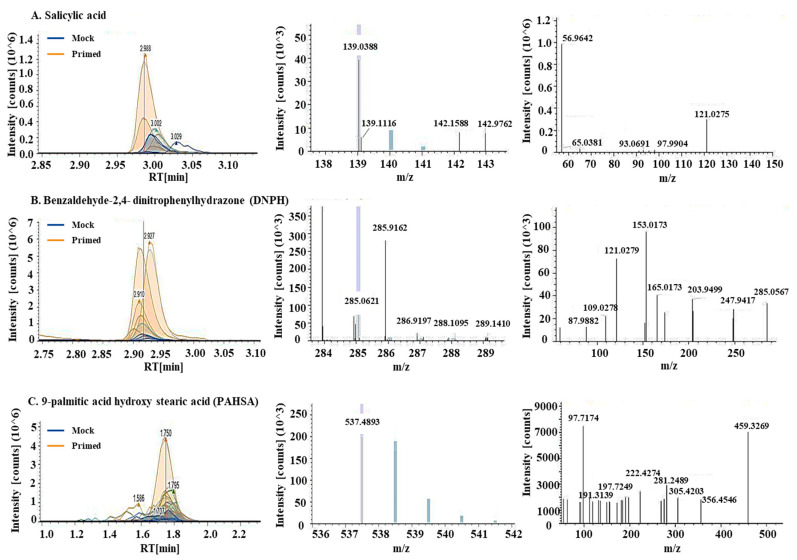
Metabolites involved in biotic stress response, lipid peroxidation, and a palmitic acid ester increased in the primed guard cells. Chromatograms, MS1, and MS2 spectra (from left to right) for (**A**) salicylic acid, (**B**) Benaldehyde-2,4-dinitrophenylhydrazone (DNPH), and (**C**) 9-palmitic acid hydroxy stearic acid (PAHSA). Chromatograms showed increases of these metabolites in the primed guard cells (orange lines) when compared to mock guard cells (blue lines). MS1 spectra for precursor ions and MS2 spectra for fragment ions allowed for quantification and identification of the metabolites.

**Figure 5:**
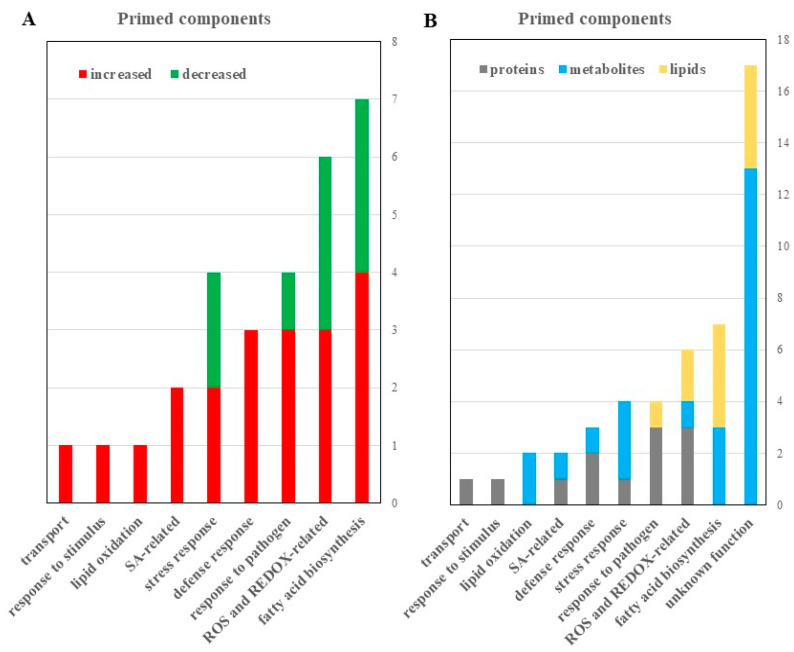
Proteomic, metabolomic, and lipidomic analyses revealed potential roles of reactive oxygen species (ROS) and fatty acid signaling in guard cell SAR. (**A**) Differential proteins, metabolites and lipids with known functions were categorized by functions and numbers that increased (red) or decreased (green) in the primed guard cells. A 2-fold change and a *p*-value of <0.05 from a student’s t-test were used. (**B**) All identified proteins (gray), metabolites (blue), and lipids (yellow) identified to be differentially abundant in primed versus mock guard cells were categorized based on functional classification.

Using untargeted metabolomics, a total of 769 metabolites were identified in the guard cell samples. Per the Metabolomics Standards Initiative [36], 689 metabolites were identified at level 2, i.e., being structurally annotated based upon their fragmentation spectral similarity with public/commercial spectral libraries. Another 80 metabolites were identified at level 3, i.e., being putatively identified based upon characteristic physicochemical properties of a chemical class, or by accurate precursor mass. After statistical analysis, 22 metabolites showed significant changes after the priming treatment (Table 1, Table S1). They are involved in defense responses (including ROS), lipid signaling and oxidation, and biosynthesis of secondary metabolites. Among the differential metabolites was SA, which was increased by 3.9-fold in the primed guard cells compared to mock samples (Figure 4A). Benzaldehyde-2,4-dinitrophenylhydrazone (DNPH), a metabolite involved in lipid peroxidation, was increased nearly 25-fold in the primed samples compared to the mock (Figure 4B). Two metabolites involved in fatty acid synthesis decreased in the primed guard cell samples compared to the mock samples. They are hexadecanedioic acid (related to palmitic acid, decreased 25-fold in primed guard cells) and decenoic acid (decreased 0.5-fold). This result indicates a role of fatty acids in guard cell priming. Thirteen differential metabolites have no known functions (Figure 5B, Table 1, Table S1).

### 2.4. Differential Lipids in the Primed and Mock Guard Cells

A total of 1197 lipids were identified in the guard cell samples. Based on the Metabolomics Standards Initiative [36], 712 were identified at level 2 and 485 were identified at level 3. After statistical analysis, 18 lipids showed significant changes in guard cells after the priming treatment (Table 1; Table S1). Their functions fall into two main categories: antioxidants and fatty acid synthesis/lipid signaling (Figure 5). Related to redox processes in the cells are two lipids: didodecyl-3,3-thiodipropionate (DLTDP) with antioxidant properties and calcitriol, a physiologically-active analog of vitamin D involved in calcium regulation [37]. Another lipid with antioxidant properties, tocopheryl acetate, was also identified as differentially abundant in primed guard cells. Related to fatty acid synthesis, two phosphatidylethanolamine (PE) lipids showed altered abundances in the primed guard cells. Additionally, both palmitic acid and the palmitic acid ester (9-palmitic acid hydroxy stearic acid (PAHSA)) increased in the primed guard cells by 2.6- and 10.6-fold, respectively, compared to the mock (Figure 4C). Palmitic acid (i.e., hexadecenoic acid) has been shown to be an agonist of Toll-like receptor 2 and 4 (TRL2 and TRL4) in animal cells. FLS2, the PRR for flagellin peptide in plant cells, shares a similar structure and function with TRL5 from human cells. If we apply a less stringent statistical criteria for significance (*p* < 0.1), 7 additional lipids were identified at level 2, and 4 at level 3. Interestingly, some of these increased lipids were also related to palmitic acid, including ethyl palmitoleate, palmitoyl ethanolamide, and oleic acid (Table S1).

## 3. Discussion

### 3.1. Significance of Guard Cell Metabolic Responses to SAR

This study highlights the importance of identifying guard cell responses to bacterial pathogen invasion because stomatal pores are the entry ports that mount a first line of defense. Typically, SAR immune responses have been studied using whole leaves [38,39,40,41], but this can mask the response of low abundant cells like guard cells, and the changes of metabolites, lipids, and proteins in these highly specialized cells can be overlooked. Also of note is that by analyzing proteins, lipids, and metabolites together, we were able to have a correlated analysis with evidence at different levels. For example, four differential proteins related to pathogen response were consistent with the increase of SA, a key regulator of pathogen response (Table 1, Table S1). In addition, the redox changes in the primed guard cells were supported by the abundance changes of two proteins, four metabolites, and two lipids with known functions related to oxidation and reduction (Table 1, Table S1). A recent report on metabolite responses to biotic and abiotic stresses focused on sample collections from whole plants, apoplastic fluids, cell walls, phloem, leaves, stems, roots, and flowers [38], but did not sample guard cells, which are known to perceive and respond to bacterial pathogens and abiotic stresses. A recent protocol by Rufian et al. [39] serves as an example of the two factors overlooked in SAR research. Firstly, they used infiltration to deliver the bacterial pathogen into apoplastic space, which completely bypasses stomatal immunity. The second is that it only focuses on the response of entire leaves, which overlooked specific responses of specialized cells like guard cells.

In leaf systemic signaling, several proteins and metabolites have been studied. For example, NPR1 is well-known as a SA receptor and is redox-regulated [42,43,44]. Cytoplasmic oligomers of NPR1 are reduced to monomers that move to the nucleus and bind with transcription factors (TFs) to activate PR genes [15,45]. Another key protein in SAR response is defective in induced resistance 1 (DIR1), a lipid transfer protein (LTP) that binds two molecules of long-chain fatty acids with high affinity [46,47,48,49]. DIR1 may enter the phloem via companion cells and act as a long-distance chaperone to systemic tissues for three other long-distance SAR signaling molecules, glycerol-3-phosphate (G3P), dehydroabietinal (DA), and azelaic acid (AzA). DIR1 interacts with AzA-induced 1 (AZI1), and both DIR1 and AZI1 are required for G3P- and AzA-induced SAR [50,51,52,53]. In addition, the presence of DIR1 and AZI1 enhanced sensitivity to DA [54,55,56]. Pipecolic acid (Pip) was found to increase during SAR, and its biosynthesis is controlled by two proteins with homologies to eukaryotic lipases, namely enhanced disease susceptibility-1 (EDS1) and phytoalexin deficient-4 (PAD4) [57,58,59,60,61]. Pip and N-hydroxypipecolic acid (NHP) have been identified as two essential metabolites for establishing SAR [62]. We may expect that some of these SAR signaling mechanisms also exist in guard cells, or alternatively that guard cells have different signaling mechanisms. Here, some similarities and differences between whole leaf SAR and guard cell SAR were identified. One similarity is the increased SA found in the primed guard cell samples compared to the mock samples. Results from leaves also showed SA increase, which is typically correlated with activation of SA biosynthesis genes [40]. Additionally, Pip and NHP also increased in uninfected leaves to amplify SA biosynthesis [40]. Wang et al. [30] observed that Pip increased at 48 h post infection (hpi) in injected leaves. It was also found in phloem exudates and increased in uninfected leaves at 48 hpi. Similarly, Bernsdorff et al. [57] reported that Pip and SA levels increased in uninfected leaves at 48 hpi. In our results, SA levels increased in systemic guard cells, but Pip did not change after initial infections. Another example of metabolic differences between leaves and guard cells appears to be the role of indolic metabolites in SAR immune response. Stahl et al. [63] found that the levels of indol-3-ylmethylamine (I3A), indole-3-carboxylic acid (ICA), and indole-3-carbaldehyde (ICC) were increased in leaves during SAR. By contrast, our guard cell samples did not show increases in these indolic metabolites. It is possible that these metabolites were not increased in guard cells but increased in other cells in the uninfected leaves after priming.

### 3.2. Increased SA in Primed Guard Cells Enhanced Systemic Defense through Stomatal Immunity

The primed guard cells in uninoculated leaves narrowed stomatal apertures and reduced entry of *Pst* into the leaves (Figure 1), indicating guard cell SAR in response to long distance immune signals. This result is consistent with a recent report [57] where decreased transpiration rates in primed *Arabidopsis* leaves were observed and related to decreases in photosynthesis-related transcripts in the primed leaves. Previously, we have identified increases in SA in primed guard cells compared to mock guard cells using targeted metabolomics [64]. Here we were able to reproduce this result using untargeted metabolomics. The increase of SA in the primed guard cells may account for the observed stomatal SAR phenotype because SA is essential for SAR [65,66]. When SA accumulation was inhibited in *NahG* plants expressing a salicylate hydroylase, SAR was abolished [13]. SA biosynthetic mutants also fail to establish SAR [67,68]. SA was reported to induce stomatal closure by activating peroxidase-mediated ROS that are integrated into a Ca^2+^/calcium protein kinase-dependent ABA signaling branch, but not the OPEN STOMATA 1 (OST1)-dependent signaling branch in *Arabidopsis* guard cells [69]. In this study, the increased levels of SA in the primed guard cells may be due to transport of methyl SA (MeSA) from mesophyll cells followed by conversion to SA [13]. Alternatively, SA could be de novo biosynthesized, since Attaran et al. [70] demonstrated that an increase in MeSA was not required for SA accumulation and SAR, and de novo synthesis of SA occurred in the leaves. In addition, MeSA appeared to be a mobile signal in dark, rather than in light [71]. Furthermore, SA could be converted from inactive glycosylated SA to induce stomatal closure.

### 3.3. Redox Changes may Mediate SAR in Guard Cells

**Figure 6:**
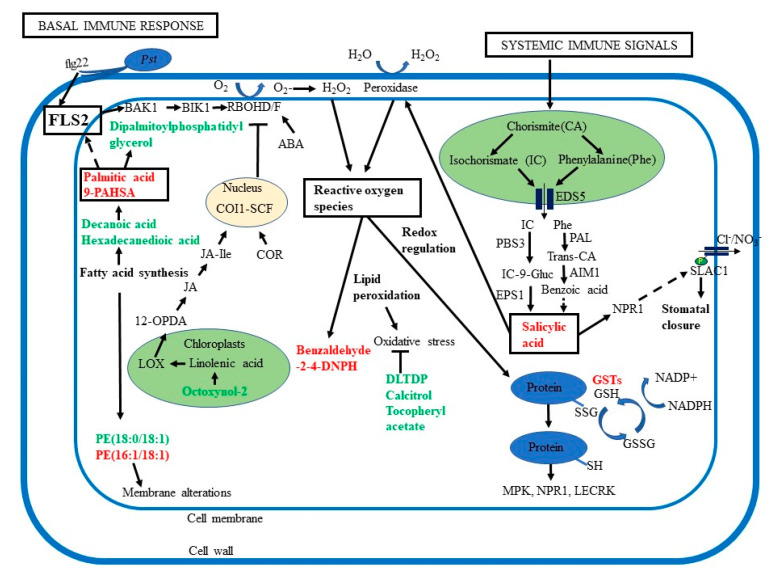
Overview of the roles of ROS, lipid signaling, and phytohormones in guard cell SAR. Guard cell SAR overlaps with basal immune response, which involves recognition of pathogen-associated molecular patterns (PAMPs) by receptors such as flagellin-sensing 2 (FLS2) and brassinosteroid insensitive 1-associated kinase 1 (BAK1), activation of Botrytis-induced kinase 1 (BIK1). BIK1 then activates respiratory burst oxidase homologs (RBOH) D/F to generate ROS. Palmitic acid, an activator for Toll-like receptors TLR 2 and 4, may also activate FLS2. Additionally, systemic immune signals activate biosynthesis of salicylic acid (SA), promoting H_2_O_2_ production via peroxidases. SA biosynthesis starts in the plastids and pathway intermediates are transported by enhanced disease susceptibility 5 (EDS5) to the cytoplasm to produce SA. SA binds to its receptor non-expressor of pathogenesis-related (NPR1), a key regulator of SAR. Abscisic acid (ABA) can also activate RBOH, adding to ROS signaling. Ultimately, both SA and ABA act on membrane anion channels, driving water to move out and stomatal closure. Primed guard cells showed decreases in octoxynol-2, related to linolenic acid, an essential fatty acid found in chloroplasts, from which 12-oxophytodienoic acid (12-OPDA), a precursor for jasmonate (JA) is derived. JA is a precursor for jasmonic acid isoleucine (JA-Ile) which binds to coronatine-insensitive 1 (COI1) and the COI1/SCF complex blocks RBOH D/F generation of ROS. During pathogen-induced stomatal opening, the *Pst* toxin coronatine (COR) structurally mimics the JA-Ile and binds to COI1. COR also represses SA synthesis, leading to reopening of stomata. Intercellular accumulation of ROS leads to oxidative stress and membrane alterations. Oxidation of fatty acids and lipids helps to reduce oxidative stress. Cellular redox perturbation regulates key signaling proteins including mitogen-activated protein kinases (MPKs), NPR1, and lectin receptor kinases (LECRK). Glutathione (GSH) is important for the glutathione redox cycle and detoxification involves GSH peroxidase, GSH reductase, and glutathione S-transferases (GSTs).

Based on the differential redox-related proteins and metabolites and lipids (Figure 5; Table 1, Table S1), we propose that the narrowed stomatal apertures may be attributed to redox changes in the primed guard cells. ROS play an important role in basal stomatal immune response that is associated with SA and changes in ion transport in the guard cells [21]. Here we show that elements of stomatal immune response overlap with systemic immunity, e.g., ROS, SA, and redox-responsive proteins (e.g., fumarase 1, ROC3, GSTF9, GSTL3, GSTU19, and GSTF7). In addition, three lipid metabolites with antioxidant properties (didodecyl-3,3-thiodipropionate, tocopheryl acetate, and calcitriol) were significantly decreased in the primed guard cells (Table 1, Figure 6, Table S1). Our results from guard cells do not match exactly the proteins and metabolites found in leaves [72], but they share similar functions as antioxidants. Excessive production of ROS causes oxidative stress and cells must ameliorate the detrimental effects of ROS. Antioxidant metabolites, such as ascorbate, glutathione (GSH), and α-tocopherol counteract stress-induced overproduction of ROS [72].

GSTs are a ubiquitous group of proteins involved in cellular redox regulation and detoxification by conjugating GSH to a variety of compounds [73,74,75]. In *A. thaliana*, the GST superfamily is composed of 53 members, which respond to different abiotic and biotic stimuli [76]. Since SA can activate transcription and translation of multiple GSTs [76], the increase of four GSTs in the primed guard cells may be attributed to the increased SA levels in the primed guard cell samples. GSTs are known to be involved in basal and systemic defense response that is redox-regulated [77]. Carella et al. [78] employed proteomic analyses from phloem taken 24 and 48 h after *Pst* inoculation of *Arabidopsis* leaves and revealed enhanced proteins that are similar to our primed guard cell results. For example, a chitinase (CHI/AED15) and a GST accumulated in the phloem during SAR, similar to the result obtained in our primed guard cells with increased chitinase family protein and GSTs (Table S1).

It is well established that SA signaling during basal defense response to pathogens is preceded by apoplastic bursts of H_2_O_2_ generated by membrane-localized NADPH oxidases and cell wall-localized peroxidases [26,79]. Cellular redox changes trigger transcriptional regulation, SA biosynthesis, and signaling, leading to programmed cell death and stomatal closure [79]. A list of *Arabidopsis* genes that have been proposed as potential ROS-mediated regulators of SA-biosynthesis include TFs that regulate isochorismate synthase 1 expression (e.g., SAR deficient 1 and WRKY8/28/48 TFs) [35,80,81], or upstream *PAD4*/*EDS1* expression (e.g., calmodulin-binding transcription activator/signal-responsive 1 and zinc-finger protein 6) [82,83]. However, we did not observe significant changes in these SA biosynthesis-related proteins in primed guard cells, indicating low abundance and/or potential posttranslational regulations.

### 3.4. Lipid Peroxidation and Signaling are Important in Guard Cell SAR

In this study, we identified lipids and fatty acids (FAs) related to lipid signaling and lipid peroxidation (LPO) that had altered abundances in primed guard cells, e.g., palmitic acid, 9-PAHSA, decanoic acid, hexadecanedioic acid, phosphorylethanolamine (PE, 18:0/18:1), and PE(16:1/18:1) (Figure 6; Table 1). ROS can induce LPO during the interaction between *Arabidopsis* and *Pst*, and LPO predominantly occurs in galactolipids and triacylglycerides proceeding programmed cell death [84]. It should be noted that this response represents a basal immune response, which induces fragmentation of galactolipids and formation of pimelic acid and AzA, a SAR mobile signal for priming [84]. ROS-mediated LPO is usually viewed as deleterious, a signal for cell death. However, it may work in parallel with the generation of reactive electrophile species (RES) in a beneficial manner for cell defense [85]. RES and jasmonates mediate oxylipins and reprogram expression of genes encoding detoxification enzymes, cell cycle regulators, and chaperones.

Our results suggest that LPO in the primed guard cells serves a beneficial function in response to SAR signals. In addition to fatty acids, we found phospholipids with altered abundances in primed guard cells. Phospholipids are structural components of membranes that can also produce signaling molecules [86]. We detected increases of palmitic acid (hexadecenoic acid) and the palmitic acid ester 9-PAHSA in the primed guard cells. Different FAs including palmitic acid (16:0), stearic acid (18:0), oleic acid (18:1), linoleic (18:2), linolenic (18:3), and palmitoleic acid (16:1) are found in plants and can be modified to form oxylipins (including JA, α-linolenic acid (18:3), and butyric acid) in plant defense. Increased levels of unsaturated 18:2 and 18:3 FAs, and decrease of 18:1 led to plant resistance to bacterial and fungal pathogens [87]. FAs also serve as precursors of phenolic lipids with antioxidant and antimicrobial properties that aid in plant defense. Some resorcinolic lipids contain SA as their phenolic group, and it is possible that free cellular SA can be derived from resorcinolic lipids [87]. Such a dynamic conversion in stomatal defense or in general plant defense has not been studied. However, our results demonstrate that increases in SA and fatty acids in primed guard cells are part of the guard cell responses during SAR.

As palmitic acid is known to activate PRR signaling pathways in human cells [88,89], the significant increases of palmitic acid and the related 9-PAHSA in guard cells (Table 1, Figure 5) may lead to activation of the plant PRR signaling via FLS2, a human TLR5 homolog. Guard cells have the PRR FLS2 that perceives the flagellin peptide of bacteria (e.g., flg22) and activates a signaling cascade involving ROS, MAP kinases, and plant hormones to close the stomata and prevent bacteria from entering the leaf apoplastic space [5,11,22,90]. Increased fatty acids in human cells lead to formation of lipid rafts and recruitment of TLRs into the lipid rafts for ligand-independent dimerization of the receptors [89]. This mechanism can allow non-ligand molecules to modulate TLR-mediated immune responses. Based on our findings, we propose that in plant guard cells palmitic acid or 9-PAHSA could play a similar role to mediate the long-distance immune response of FLS2. Although our data have laid a foundation for this new hypothesis, further experiments need to be conducted to test the hypothesis. In addition to compounds such as palmitic acid and 9-PAHSA with known functions, we also identified 40 metabolites and lipids with unknown functions (Figure 5B, Table 1, Table S1). These compounds provide an important resource for the community, and they could be used for follow-up pharmacological studies to determine guard-cell defense response and aid in the identification of crop protection strategies [91].

## 4. Materials and Methods

### 4.1. Plant Growth and Bacterial Culture

*A. thaliana* Col-0 seeds were obtained from Arabidopsis Biological Research Center (Columbus, OH, USA). They were suspended in deionized H_2_O and vernalized at 4 °C for two days before planting. The seeds were cultivated in soil and grown in controlled environmental chambers in short day (8-h light/16-h dark) environment. The temperatures during the light and dark periods were 22 °C and 20 °C, respectively. Incandescent bulbs capable of emitting 140 µmol m^−2^·s^−1^ at the leaf surface were used in the growth chambers with a relative humidity of about 60%. A dome was placed over the flat until seeds began germination. After 2 weeks of growth, seedlings were transferred into individual pots. Plants were watered weekly, kept in the chambers until mature rosette (stage 3.9), and observed at 5 weeks of age [92].

*Pseudomonas syringae* pv. *tomato* DC3000, the model pathogen for *Arabidopsis* SAR induction [22,39], was used for the experiments. Agar media plates were made using King’s B media protocol. A 1-L solution was made, containing 20 g Protease peptone No. 3, 1.5 g K_2_HPO_4_ (s), 0.75 g MgSO_4_ (s), 10 mL glycerol, 15 g agar, and deionized H_2_O King’s B Media was autoclaved, and antibiotics Rifampicin (25 mg/L) and Kanamycin (50 mg/L) were added once the solution was cooled. Solution with agar was used for plates and *Pst* colonies were streaked on this medium and incubated for overnight at 28 °C. *Pst* colonies were grown in the same King’s B media without agar in solution overnight, pelleted by centrifugation, and used for treatment of *Arabidopsis* plants.

### 4.2. Stomata Aperture Measurements

Primary inoculation occurred via needleless syringe injection, where the plants were either primed with *Pst* DC3000 (OD_600_ = 0.02) suspended in 10 mM MgCl_2_ or mock-treated with 10 mM MgCl_2_. At 3 days post inoculation, the leaf opposite to the injected leaf was detached for a secondary treatment. In the secondary treatment, the leaves were either floated in 10 mM MgCl_2_ or in *Pst* DC3000 (OD_600_ = 0.2) solution in small petri-dishes. Three leaves were used for each time point and secondary treatment group, and only one leaf was collected from each plant. Stomatal apertures were measured at three time points: 0 h, 1 h, and 3 h. At each time point, the leaves were collected and peeled using clear tape. The abaxial side of the leaf was then placed on a microscope slide and images were collected using confocal microscopy. This experiment was repeated 3 times to image 50 stomata from each replicated treatment and a total of 150 stomata measurements from 3 independent replicates were analyzed for each time point. Stomatal apertures were measured using ImageJ software (National Institutes of Health, Bethesda, MD, USA).

### 4.3. Pst DC3000 Entry and Growth Assays

To measure how much bacteria entered the apoplast after 3 h, 9 independent biological replicates of *Arabidopsis* plants were grown to 5 weeks and prime-treated via injection with either *Pst* DC3000 (OD_600_ = 0.02) or mock-treated with 10 mM MgCl_2_. Three days after the infection, the leaf opposite to the one infected was detached and floated in *Pst* (OD_600_ = 0.2) solution for both mock and primed plants. After three hours of floating in solution, leaf was placed in Falcon tube with 0.02% Silwet, vortexed for 10 s, dried with sterile Kim wipes, wrapped in clean aluminum foil, and taken to Laminar flow hood for aseptic treatment. In the hood, an autoclaved hole-puncher was used to obtain one disk from each leaf (0.5 cm diameter), and the disk was placed in 100 µL sterile H_2_O. Each leaf disk was then ground using an autoclaved plastic grinding tip, and 10 µL of the solution was collected to make a 1:1000 serial dilution. From the dilution, 100 µL was collected and plated on agar media containing Rifampicin (25 mg/L) and Kanamycin (50 mg/L). After 2 days of incubation at 28 °C, the colonies on the plate were counted. The experiment was repeated 3 times with 3 replicates of each treatment and the bacterial counts of 9 replicates were used to calculate mean and standard error.

*Pst* growth experiment determines how much bacteria grow in the apoplast after 3 days. *Arabidopsis* plants (9 independent replicates) were grown to 5 weeks and either mock or prime-treated. After three days of treatment, the upper and lower rosette leaves were sprayed with *Pst* DC3000 (OD_600_ = 0.2) and a dome was put on top for 24 h. After 24 h, the dome was removed and the plants were left in growth chamber for another 48 h. One opposite leaf of each plant was then detached and washed in 0.02% Silwet, and one disk was taken from leaf to make a 1:1000 serial dilution and plate it on media. Colonies were counted to determine how much bacteria were able to grow in the apoplast. The experiment was repeated 3 times with 3 replicates of each treatment and the bacterial counts of 9 replicates were used to calculate mean and standard error.

### 4.4. Isolation of Enriched Guard Cells for Multi-omics Experiments

Enriched guard cell samples were prepared as previously described [64]. Briefly, for each sample, 144 upper uninfected leaves were collected from 36 individual plants (i.e., four upper primed leaves per plant). After removing the midvein with a scalpel, the leaves were blended for 1 min in a high-speed blender with 250 mL of distilled deionized water and ice. The sample was then filtered through a 200 µm mesh filter. This process was repeated 3 times to obtain intact guard cells, which were collected immediately into 15 mL Falcon tubes, snap frozen in liquid nitrogen, and stored in −80 °C.

Freshly collected guard cells were resuspended in 1 mL of deionized distilled water in a 1.5 mL tube, then 100 μL of 0.03% neutral red working solution was added. The tube was gently mixed and incubated at room temperature for 5 min. Samples were spun down and washed twice with deionized distilled water. After washing, samples were observed on a light microscope (Leica DM 6000 B, Buffalo Grove, IL, USA) for imaging. Viable cells accumulate neutral red in the vacuoles, thus are stained red. Ten images of 174 guard cells were randomly selected and guard cells were scored for viability. This process was repeated for staining with non-polar fluorescein-diacetate (FDA). The procedure is the same as the neutral red staining, except 100 μL 2.5 μM FDA was added to the guard cells. After washing with deionized distilled water, fluorescence microscopy was used to image the cells using a filter cube that allows an excitation wavelength of around 460 nm, and an emission wavelength of around 525 nm (e.g., with a 450–490 nm bandpass excitation filter and a 500–550 nm bandpass emission filter) (Leica DM 6000 B, Buffalo Grove, IL, USA). Images were obtained under both bright field and fluorescence excitation. FDA enters live cells and is hydrolyzed by esterases in the cell producing fluorescein. From the FDA staining, 10 images of 134 guard cells were randomly selected for viability assay. Those that contained the FDA fluorescence were counted as viable and those that did not were counted as non-viable. Based on both neutral red and FDA results, the guard cell viability was 80%.

### 4.5. Chlorophyll Assay and RT-qPCR of Guard Cells and Leaves

Chlorophyll was quantified from leaves and enriched guard cells as previously described [93]. Briefly, four replicates of leaves or guard cells of mock or primed plants were obtained with four mature leaves in each replicate. Samples were frozen in liquid nitrogen and then ground to a fine powder using a mortar and pestle. Material fresh weight (FW) was weighed, and 1.5 mL of 80% (*v*/*v*) acetone was added, vortexed, and placed in dark for 30 min. After centrifugation at 13,000 g for 15 min to pellet debris, the supernatant was removed and measured at 663 nm (chlorophyll *a*) and 646 nm (chlorophyll *b*) using a spectrophotometer. Total chlorophyll *a* and *b* was determined using the following formulae: (12.7 × A_663_ − 2.69 × A_646_) × volume/weight = Chl *a* mg/g FW and (22.9 × A_646_ − 4.86 × A_663_) × volume/weight = Chl *b* mg/g FW.

To determine the expression level of guard cell specific genes in different samples, RNeasy Plant Mini Kit (QIAGEN, Germantown, MD, USA) was used for total RNA extracting. PrimeScript RT reagent Kit (New England Biolabs, Ipswich, MA, USA) was used for cDNA synthesis. The 2× SYBR Green qPCR Master Mix kit (Bimake, Houston, Texas, USA) was used for qRT-PCR with a CFX96 171 Touch™ Real-Time PCR Detection System (Bio-Rad, Hercules, CA, USA). Three biological replicates and three technological replicates were carried out for each sample. Relative expression levels of the genes were analyzed using the 2^−△△*C*t^ method. *ACTIN2* was used as the internal reference (Figure 2A). The real-time primers used are as follows: *OST1* F: CGATAACACGATGACCACTC, *OST1* R: CCAAGCTTCCTGTGAGGTAA; *GORK* F: GATCACCGCTTCATCTTGCAG, *GORK* R: TTGTCCTGCTTTCACAGCCT; *HT1* F: ATCGTTCAGTTCATTGCGGC, *HT1* R: GGTCTCGATCGAAAGCGAGT; *KAT1* F: CCTCTCTGCCGATCTTCTACC, *KAT1* R: CACATCTCCCACGCTCTGTA; *PYL2* F: AAGCGTCAGAGAAGTGACCG, *PYL2* R: AGAACACGGTGGTCGTCATC; *PGC* F: TTCTCAGGTACTTGCAGAGTTGTC, *PGC* R: GGTTCTCGTCATTCGTCTGAG; *ACTIN2* F: CGTACAACCGGTATTGTGCTG, *ACTIN2* R: AGTAAGGTCACGTCCAGCAAG.

### 4.6. 3-in-1 Protein, Metabolite, and Lipid Extraction Method

A biphasic methanol and chloroform method adapted from Bligh and Dyer [94] was used to obtain proteins, metabolites, and lipids from 100 mg of freeze-dried guard cells harvested from the mock and primed plants. Guard cell samples were quickly immersed in glass tubes with 3 mL pre-heated 75 °C methanol (MeOH) and 0.01% butylated hydroxytoluene (BHT) for 15 min. Internal standards were added for proteins: 60 fmol digested bovine serum albumin (BSA) peptides per 1 μg sample protein; for metabolites: 10 μL 0.1 nmol/μL lidocaine and camphorsulfonic acid; and for lipids: 10 μL 0.2 μg/μL deuterium labeled 15:0–18:1(d7) phosphatidylethanolamine (PE) and 15:0–18:1(d7) diacylglycerol (DG). For extraction, 6 mL chloroform and 2 mL water (3:1, *v*/*v*) were added to each tube, then 500 μL MeOH was added to replenish the methanol that evaporated during boiling. Samples were vortexed and then agitated at 4 °C for 1 h. For phase separation, extracts were centrifuged for 10,000 rpm for 10 min at 4 °C. The upper (metabolite in MeOH) phase was collected into the plastic 2 mL centrifuge tubes and the bottom (lipid in chloroform) phase into glass tubes, leaving the middle (protein) layer for further protein collection. To improve component collection, 2 mL chloroform/methanol (2:1 *v*/*v*) with 0.01% BHT was added again and then the tubes were shaken for 30 min at 4 °C. Extracted liquid was combined into the glass centrifugal tubes and the extraction procedure was repeated one additional time to obtain complete extraction from tissue samples. The lipid extracts were dried under nitrogen gas to prevent oxidation and stored in −80 °C. The lipid extract was later dissolved in 1 mL isopropanol for LC-MS/MS analysis. Aqueous metabolites were lyophilized and placed at −80 °C. Aqueous metabolite pellets were later solubilized in 100 μL 0.1% formic acid in H_2_O for LC-MS/MS analysis. Protein components were collected by precipitation in cold 80% acetone in the centrifuge tubes at −20 °C overnight.

### 4.7. Protein Extraction, Digestion, and LC-MS/MS

Three biological replicates of mock and SAR primed guard cell samples were prepared for proteomic experiments. Proteins were precipitated by addition of 80% acetone in glass centrifuge tubes in −20 °C overnight. Acetone was removed using glass pipettes, and the tubes with the protein samples were dried in a speedvac. Protein samples were then resuspended in 50 mM ammonium bicarbonate, reduced using 10 mM dithiothreitol (DTT) at 22 °C for 1 h, alkylated with 55 mM indol-3-acetic acid (IAA) in darkness for 1 h, then digested with trypsin for 16 h.

MS data acquisition was performed using an Easy-nLC coupled to a Q Exactive hybrid quadrupole-Orbitrap MS/MS system (Thermo Scientific, Bremen, Germany) with a nanoelectrospray ion source. Sample peptides (10 µL volume) were injected onto an Acclaim PepMap™ 100 pre-column (75 µm × 2 cm, nanoViper C18, 3 µm, 100 A) and separated in an Acclaim PepMap™ RSLC (75 µm × 25 cm, nanoViper C18, 2 µm, 100 A) analytical column with a linear gradient of solvent B (0.1% formic acid, 99.9% Acetonitrile) from 1% to 30% for 90 min at 250 nL/min. The MS was operated between MS scan and MS/MS scan automatically with a cycle time of 3 s. Eluted peptides were detected in the Orbitrap MS at a resolution of 120 K with a scan range of 350–1800 *m*/*z*, and the most abundant ions bearing 2–7 charges were selected for MS/MS analysis. Automatic gain control (AGC) for the full MS scan was set as 200,000 with maximum injection time (MIT) as 50 ms, and AGC target of 10,000 and MIT of 35 ms were set for the MS/MS scan. The MS/MS scan used quadrupole isolation mode, collision-induced dissociation (CID) activation energy, 35% collision energy, and an IonTrap detector. A dynamic exclusion time of 30 s was applied to prevent repeated sequencing of the most abundant peptides.

### 4.8. Metabolite, Lipid Preparation, and LC-MS/MS

The untargeted metabolomic approach used the high resolution Orbitrap Fusion Tribrid mass spectrometer (Thermo Fisher Scientific, Waltham, MA, USA) with Vanquish™ UHPLC liquid chromatography. An Accucore C18 (100 × 2.1) column was used for metabolites with solvent A (0.1% formic acid in water) and solution B (0.1% formic acid in acetonitrile). The column chamber temperature was to 55 °C. Pump flow rate was set to 0.45 mL/min. The LC gradient was set to 0 min: 1% of solvent B (i.e., 99% of solvent A), 5 min: 1% of B, 6 min: 40% of B, 7.5 min: 98% of B, 8.5 min: 98% of B, 9 min: 0.1% of B, 10 min stop run. To enhance identification, an Acquire X MSn data acquisition strategy was used which employs replicate injections for exhaustive sample interrogation and increases the number of compounds in the sample with distinguishable fragmentation spectra for identification. Electrospray ionization (ESI) was used in both positive and negative modes with a spray voltage for positive ions (V) = 3500 and a spray voltage for negative ions (V) = 2500. Sheath gas was set to 50, auxiliary gas was set at 1 and sweep gas was set to 1. The ion transfer tube temperature was set at 325 °C and the vaporizer temperature was set at 350 °C. Full MS1 used the Orbitrap mass analyzer (Thermo Fisher Scientific, Waltham, Massachusetts, USA) with a resolution of 120,000, scan range (*m*/*z*) of 55–550, MIT of 50, AGC target of 2e^5^, 1 microscan, and RF lens set to 50%.

For untargeted lipidomics, a Vanquish HPLC-Q Exactive Plus system was used with an Acclaim C30 column (2.1 mm × 150 mm, 3µm). Solution A for lipids consisted of 0.1% formic acid, 10 mM ammonium formate, and 60% acetonitrile. Solution B for lipids consisted of 0.1% formic acid, 10 mM ammonium formate, and 90:10 acetonitrile: isopropyl alcohol. The column chamber temperature was set to 40 °C. Pump flow rate was set to 0.40 mL/min. The LC gradient was set to 0 min: 32% of solvent B (i.e., 68% of solvent A), 1.5 min: 45% of B, 5 min: 52% of B, 8 min: 58% of B, 11 min: 66% of B, 14 min: 70% of B, 18 min: 75% of B, 21 min: 97% of B, 26 min: 32% of B, 32 min stop run. The method for Q Exactive Plus mass spectrometer included a 32-min duration time, 10 s chromatogram peak width with full MS and ddMS^2^. Ion fragmentation was induced by CID, with positive polarity and a default charge state of 1. Full MS1 used the Orbitrap ion trap mass analyzer with a resolution of 70,000, 1 microscan, AGC target set to 1e^6^, and a scan range from 200 to 2000 *m*/*z*. The dd-MS² scan used 1 microscan, resolution of 35,000, AGC target 5e^5^, MIT of 46 ms, loop count of 3, isolation window of 1.3 *m*/*z*, and a scan range of 200 to 2000 *m*/*z* for positive and negative polarity.

### 4.9. Data analysis for Proteins, Metabolites, and Lipids

For LC-MS/MS proteomic data analysis, we used Proteome Discoverer™ 2.4 (Thermo Fisher Scientific, Waltham, MA, USA) with the SEQUEST algorithm to process raw MS files. Spectra were searched using the TAIR10 protein database with the following parameters: 10 ppm mass tolerance for MS1 and 0.02 da as mass tolerance for MS2, two maximum missed tryptic cleavage sites, a fixed modification of carbamidomethylation (+57.021) on cysteine residues, dynamic modifications of oxidation of methionine (+15.996) and phosphorylation (+79.966) on tyrosine, serine, and threonine. Search results were filtered at 1% false discovery rate (FDR) and peptide confidence level was set for at least two unique peptides per protein for protein identification.

Relative protein abundance in primed and control guard cell samples was measured using label-free quantification in Proteome Discoverer™ 2.4 (Thermo Scientific, Bremen, Germany). Proteins identified and quantified in all 3 out of 3 sample replicates were used, and no imputation was performed. Peptides in mock and primed samples were quantified as area under the chromatogram peak. Peak areas were normalized by the bovine serum albumin (BSA) internal standard added during sample preparation and extraction. The average intensity of three primed vs. three mock samples were compared as a ratio and two criteria were used to identify significantly altered proteins: (1) increase of 1.2 or decrease of 0.8 (prime/mock), and (2) *p*-value from an unpaired Student’s *t*-test less than 0.05. 

For untargeted metabolomics, Compound Discover™ 3.0 Software (Thermo Scientific, Bremen, Germany) was used for data analyses. Raw files from three replicates of mock and three replicates of primed guard cells were used as input. Spectra were processed by aligning retention times. Detected compounds were grouped and gaps filled. Peak area was refined from normalize areas while marking background compounds. Compound identification included predicting compositions, searching *mz*Cloud spectra database, and assigning compound annotations by searching ChemSpider, pathway mapping to KEGG pathways and to Metabolika pathways was included for functional analysis of the metabolites. The metabolites were scored by applying *mz*Logic and the best score was kept. Peak areas were normalized by the positive and negative mode internal standards (lidocaine and camphorsulfonic acid, respectively) added during sample preparation. For untargeted lipidomics data analyses, Lipid Search 4.1™ and Compound Discover™ 3.0 (Thermo Scientific, Bremen, Germany) were used. Raw files from three replicates of mock and three replicates of primed guard cells were uploaded Lipid Search 4.1™ for annotation of lipids found in all the samples. A mass list was generated for uploading to Compound Discover™ 3.0 Software. This mass list was used for compound identification along with predicted compositions, searching mzCloud spectra database, and assigning compound annotations by searching ChemSpider. Peak areas were normalized by median-based normalization. For both metabolomics and lipidomics, the average areas of three primed vs. three mock metabolite samples were compared as a ratio and two criteria were used to determine significantly altered metabolites or lipids: (1) increase of 1.2 or decrease of 0.8 (prime/mock), and (2) *p*-value from an unpaired Student’s *t*-test less than 0.05. 

## 5. Conclusions

In conclusion, our work here makes a clear distinction from previous studies in that it specifically targets the guard cell SAR. Our results have shown: (1) guard cells have specific metabolic responses to long-distance SAR priming that are different from leaves; (2) directly measured increased SA in primed guard cells leading to narrowed stomata aperture, and reduced bacterial entry and growth in leaves; (3) increased SA, redox changes, lipid peroxidation, and signaling may mediate the guard cell SAR; and (4) palmitic acid and its ester 9-PAHSA may function as activators of FLS2 in primed guard cells. Identifying lipid, metabolite, and protein components of guard cell SAR is a critical first step in analyzing how stomatal immunity plays a role in plant defense. Much additional work still needs to be done, e.g., functional testing of the newly identified components in guard cell SAR using reverse genetics. Additionally, a pharmacological approach could be taken to confirm the effectiveness of palmitic acid and 9-PAHSA in stomatal immune responses. Another route for future experimentation may involve transcriptomics toward comprehensive understanding of the transcriptional regulation in guard cell SAR.

## Figures and Tables

**Table 1:** List of proteins (top), metabolites (middle), and lipids (bottom) with more than two-fold changes in guard cells after systemic acquired resistance (SAR) priming. (The protein IDs are from TAIR database and the compound IDs are from PubChem. The ratios are Primed/Mock).

Protein/Compound	ID	*p*-Value	Ratio	Biological Function
ROC-rotamase CYP3	AT2G16600	0.03	2.52	ROS, response to stimulus/pathogen
Glutathione S-transferase (GST) 7	AT1G02920	0.004	8.63	ROS, defense response, response to stimulus/pathogen
Endoribonuclease L-PSP family protein	AT3G20390	0.03	4.09	Isoleucine biosynthesis, response to stimulus/stress
Salicylic acid	338	0.02	3.85	Defense response, biosynthesis of antibiotics
Decenoic acid	5282724	0.02	0.20	Lipid signaling, biosynthesis of fatty acids/metabolites
Hexadecanedioic acid	10459	0.02	0.04	Lipid signaling/oxidation, related to fatty acids
Benzaldehyde-2,4-DNPH	9566364	0.003	25.33	Lipid signaling, lipid peroxidation
4-Oxo-4-(3-oxo-3,4-dihydro-1(2H)-quinoxalinyl) butanoic acid	3146205	0.03	3.02	Lipid oxidation, fatty acid synthesis
N-(1,3-benzodioxol-5-ylmethyl)-2-methyl-6-(trifluoromethyl) nicotinamide	2811293	0.04	3.63	Stress response, protection against cell leakage/DNA damage
5-Methyl-7-phenyl-6,7-dihydro-1H-1,4-diazepine-2,3-dicarbonitrile	2763570	0.03	0.06	Stress response, biosynthesis of secondary metabolites, ubiquinone and terpenoid-quinone
Octoxynol	24775	0.02	0.28	Stress response, linolenic acid/hormone metabolism
Didodecyl-3,3-thiodipropionate (DLTDP)	31250	0.047	0.14	Oxidation-reduction, antioxidant
Pentaethylene glycol-n5	62551	0.008	0.21	Unknown function
NP-015468	-	0.03	0.16	Unknown function
Triphenylphosphine oxide	13097	0.04	0.20	Unknown function
Di-tert-butyl dicarbonate	90495	0.03	0.18	Unknown function
{1-Methyl-6-[(1-methyl-1H-benzimidazol-2-yl)methyl]-5-oxodecahydropyrrolo[1,4] diazepin-2-yl}-N-(2-thienylmethyl)propanamide	-	0.04	0.14	Unknown function
1792084-C15H29NO3	-	0.02	0.29	Unknown function
Sym-triaminotrinitrobenzene	18286	0.002	0.30	Unknown function
[4,4′-Bipyridine]-3,5-dicarbonitrile, 2,6-dihydroxy	95562431	0.02	0.27	Unknown function
Isoxazolecarboxylic acid, 5-methyl-, 2-benzyl-2-(5-methyl-3-isoxazolylcarbonyl)hydrazide	5334020	0.03	3.22	Unknown function
2-Propenoic acid, 2-methyl-, oxydi-2,1-ethanediyl ester	16891	0.01	0.18	Unknown function
1-methyl-N′-[(E)-(4-nitrophenyl)methylidene]-6-oxo-1,6-dihydro-3-pyridinecarbohydrazide	-	0.03	3.22	Unknown function
6,7-Benzomorphan	182394	0.02	3.06	Unknown function
Calcitriol	5280453	0.04	0.15	Oxidation-reduction, vitamin D, calcium regulation
Ergosterol	444679	0.04	0.2	Response to pathogen, antifungal, membrane integrity, biosynthesis of metabolites
PE(18:0/18:1(11Z))-(2R)-3-{[(2-aminoethoxy) (hydroxy)phosphoryl]oxy}-2-(pentadecanoyloxy)propyl stearate	9547031	0.03	0.37	Lipid signaling or oxidation, fatty acid synthesis, biosynthesis of phosphatidylcholine
PE(16:1(9Z)/18:1(11Z))-(2R)-3-{[(2-Aminoethoxy)(hydroxy) phosphoryl]oxy}-2-[(9Z,12Z,15Z)-9,12,15- octadecatrienoyloxy] propyl (15Z)-15-tetracosenoate		0.04	2.31	Lipid signaling or oxidation, fatty acid synthesis
Palmitic acid (hexadecanoic acid)	985	0.02	2.63	Lipid signaling/oxidation, fatty acid synthesis, secondary metabolites, lipid rafts
9-PAHSA (9-palmitic acid hydroxystearic acid)	72189985	0.03	10.59	Lipid signaling or oxidation, lipid rafts
3-[(11E,15E)-11,15-Dotriacontadien-1-yl]-5-methyl-2(5H)-furanone	101949817	0.03	0.50	Unknown function
Dodecanamide, N,N′-1,8-octanediylbis	3273664	0.047	0.49	Unknown function
[4,4′-Bipyridine]-3,5-dicarbonitrile, 2,6-dihydroxy	95562431	0.02	0.27	Unknown function
3,5-Dibromoisonicotinonitrile	42553006	0.02	0.43	Unknown function

## Data Availability

The proteomics data have been deposited to the ProteomeXchange Consortium via the PRIDE partner repository with the data set identifier PXD022880, and the metabolomics data have been deposited to the MetaboLights data repository with the data set identifier MTBLS2261.

## Supplementary Materials

The following is available online at https://www.mdpi.com/1422-0067/22/1/191/s1. Table S1: Detailed information on proteins, metabolites and lipids identified and quantified in this study.

## Abbreviations

SAR	Systemic acquired resistance
ROS	Reactive oxygen species
FA	Fatty acids
LPO	Lipid peroxidation
